# The Impact of COVID-19 Pandemic on Hospitalization and Interventional Procedures for Cardiovascular Diseases during the First Wave in Italy

**DOI:** 10.3390/ijerph20010472

**Published:** 2022-12-28

**Authors:** Vincenzo Russo, Luigi Cante, Egidio Imbalzano, Pierpaolo Di Micco, Roberta Bottino, Andreina Carbone, Antonello D’Andrea

**Affiliations:** 1 Cardiology Unit, Department of Translational Medical Sciences, University of Campania “Luigi Vanvitelli”, Monaldi Hospital, 80131 Naples, Italy; 2 General Medicine, Thrombotic and Hemorrhagic Unit, Department of Internal Medicine, University of Messina, 98122 Messina, Italy; 3 Emergency Department, Rizzoli Hospital, Health Authority NA2, Ischia, 80122 Napoli, Italy; 4 Cardiology and Intensive Coronary Care Unit, “Umberto I” Hospital, 84014 Nocera Inferiore, Italy

**Keywords:** COVID-19, pandemic, cardiovascular disease, implanted devices, ablation, syncope, acute coronary syndrome, heart failure, drug adherence, telemedicine

## Abstract

Coronavirus disease 2019 (COVID-19) has been responsible for an epidemic of devastating proportion, and it has represented a challenge for worldwide healthcare systems with the need of resources reallocation in order to face epidemic spread. Italy was one of the hardest hit countries by COVID-19, and the Italian government adopted strict rules to contain the spread of the COVID-19 pandemic, such as national lockdown and home quarantine; moreover, the Italian healthcare system had to rapidly re-organize the diagnostic and therapeutic pathways, with a reallocation of health resources and hospital beds, in order to manage COVID-19 patients. The aim of the present review is to provide an overview of the effects of the first pandemic wave on cardiovascular assistance in Italy with the purpose of evaluating the strengths and weaknesses of the Italian health system.

## 1. Introduction

Coronavirus disease 2019 (COVID-19) is the infectious disease caused by the severe acute respiratory syndrome coronavirus 2 (SARS-CoV-2), a highly pathogenic human coronavirus responsible for an epidemic of devastating proportion (over 650 million cumulative cases and over 6 million cumulative deaths worldwide) [1,2]. In Italy, there were about 24,000,000 confirmed cumulative cases and more than 180,000 cumulative deaths [3]. The Italian government adopted strict rules to contain the spread of the COVID-19 pandemic, such as national lockdown and home quarantine; moreover, the Italian Healthcare System had to rapidly re-organize diagnostic and therapeutic pathways, with a reallocation of health resources and hospital beds, in order to manage COVID-19 patients. Several studies described a reduction in hospitalizations and interventional procedures for cardiovascular disease (CVD) across different regions of Italy during the first pandemic wave; however, overall descriptive data has not yet been provided. The aim of this review is to describe the effects of the first COVID-19 pandemic wave on cardiovascular assistance in Italy with the purpose to underline strengths and weaknesses of Italian Healthcare System.

## 2. Cardiac Pacing Procedures

**Figure 1:**
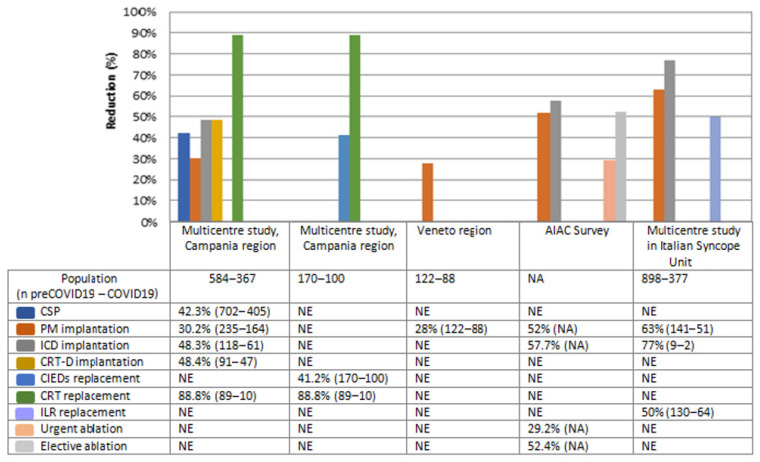
The graph shows the comparison between hospitalization rate relating to CSP in the COVID-19 era and those relating to a previous control period in different Italian hospitals. Reported data refer to Campania region [4,5], Veneto region [6], AIAC survey [7] and a multicentre study from Italian Syncope Units [8]. Each cell shows the percentage reduction in hospitalizations for a specific procedure compared to the pre-pandemic period, followed respectively by the absolute number of procedures in the pre-COVID 19 and COVID 19 era (data in brackets). CSP: Cardiac Stimulation Procedures. PM: Pacemaker. ICD: Implantable Cardioverter Defibrillator. CRT-D: Cardiac Resynchronization Therapy Defibrillator. ILR: Implantable Loop Recorder. NE: Not evaluated. NA: Not available.

Several observational studies described the impact of the COVID-19 outbreak on cardiac pacing and electrophysiology activities during the first lockdown in Italy (Figure 1).

A retrospective multicenter study [4], carried out in Campania region, the most-populous region of Southern Italy with about 5.8 million residents, showed a significant reduction in the rate of all cardiac implantable electronic devices (CIEDs) implantations and in cardiac resynchronization therapy (CRT) and device replacements (*p*-value < 0.05), more likely because of the reduction in planned hospitalizations following Italian government measures to contain SARS-CoV-2 in-hospital diffusion. No significant differences in both pacemaker (PM) and implantable cardiac defibrillator (ICD) replacement procedures (*p*-value 0.297 and 0.076, respectively) have been shown, more likely because of the increase in urgent unplanned hospitalizations. The increased use of remote monitoring (RM) to optimize the hospital admission time during COVID-19 lockdown for CIEDs recipients may explain these data [5,9].

No difference in the admission rate at Emergency Departments for patients in need of cardiac pacing was shown, in particular for those who experienced syncope [4,10]. This latter evidence contrasts with reports from Northern Italy hospitals, which showed a reduction in both temporary and definitive PM implantations in emergency conditions in Veneto region and Trieste city, that have 4.9 and 0.2 million inhabitants, respectively [6,11]. It might be possible that the lower epidemiological pressure of COVID-19 in Campania region compared to the majority of the regions in Northern Italy (positive cases up to 13 times higher), influenced these results.

## 3. Electrophysiology Procedures

An Italian national survey involving 104 physicians from 84 Italian arrhythmia centers [7] reported a remarkable reduction of ablation procedures, including those performed in emergency settings, such as ablations of electrical storm, refractory ventricular tachycardia (VT), or supraventricular tachycardia (SVT), that require high competence and usually cannot be deferred. A single-center, retrospective, observational study, conducted at an Italian third-level electrophysiology laboratory, reported a drastic decline in ablation procedures for SVT, atrial flutter and atrial fibrillation compared to those performed in pre-COVID 19 period [12].

## 4. Syncope Unit Activities

A retrospective multicenter observational study including 10 Italian Syncope Units (SUs), certified by the Italian Multidisciplinary Working Group on Syncope (GIMSI), suggested that COVID-19 lockdown was associated with a significant reduction rate in all the clinical activities related to the differential diagnosis of transient loss of consciousness (from 141 to 51 (−63%), *p*-value 0.001). The remarkable changes in the third-level SUs activities were more likely due to the reduction of patients referred from other hospital wards, which were converted into COVID-19 care centers [8].

## 5. Acute Coronary Syndrome

**Figure 2:**
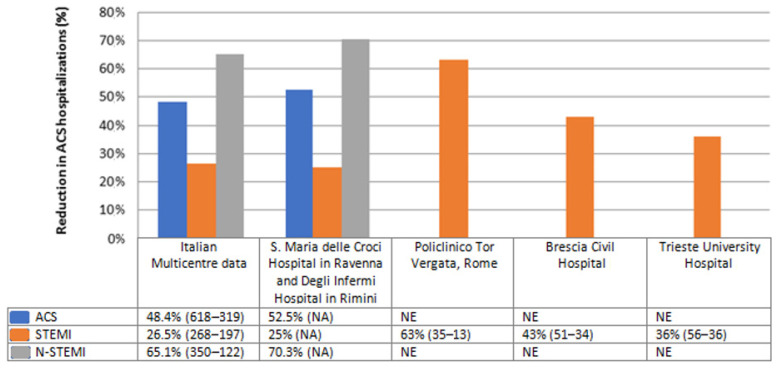
The graph shows the comparison between data relating to ACS hospital admissions in the COVID-19 era and those relating to a previous control period in different Italian hospitals. Reported data refer to Italian Multicentre study [13], a study performed in S. Maria delle Croci Hospital in Ravenna and Degli Infermi Hospital in Rimini [14], Policlinico Tor Vergata [15], Brescia Civil Hospital [16] and Trieste University Hospital [17]. Each cell shows the percentage reduction in hospital compared to the pre-pandemic period, followed respectively by ACS absolute number in the pre-COVID 19 and COVID 19 era (data in brackets). ACS: Acute Coronary Syndrome. STEMI: ST-Elevation Myocardial Infarction. N-STEMI: Non-ST-Elevation Myocardial Infarction. NE: Not evaluated. NA: Not available.

Some observational studies described a significant reduction (*p*-value < 0.05) of acute coronary syndrome (ACS) hospitalizations (Figure 2) with delays in urgent care and an increased rate of arrhythmic and mechanical complications. 

A multicenter, observational, nationwide survey [13] included 319 consecutive patients with acute myocardial infarction (AMI) admitted to Italian intensive cardiac care units (CCUs) throughout a one-week period during the COVID-19 outbreak in Italy. A 48.4% reduction in the number of ACS hospitalizations, a 26.5% reduction in ST-Elevation Myocardial Infarction (STEMI) admissions and a 65.1% reduction in non-ST-Elevation Myocardial Infarction (N-STEMI) admissions has been shown compared to the same period in 2019. The overall reduction was similar nationwide (northern Italy (52.1%); central Italy (59.3%) and southern Italy (52.1%)).

Several observational data from Italian high-volume referral centers confirmed this trend (Image 2), reporting a reduction in ACS admissions ranging from 48.4% [13] to 52.5% [14]; in STEMI admissions from 25% [14] to 63% [15], and a reduction in N-STEMI admissions from 65.1% [13] to 70.3% [14]. The number of ACS-related complications (cardiogenic shock, life-threatening arrhythmias, post-ischemic interventricular defect, post-ischemic mitral regurgitation) and the mortality rate (13.7% in 2020 versus 4.1% in 2019) was higher than in the pre-COVID era [13,16]. These data may be explained by the late medical presentations (subacute myocardial infarction) [18] and admission to catheter laboratory [19]. During the COVID-19 outbreak, the observed higher values of cardiac biomarkers at the time of hospital admission, higher prevalence of STEMI patients with TIMI flow ≤ 2, and greater use of glycoprotein IIb/IIIa inhibitors are probably linked with the patient’s late admission at the Emergency Department. Indeed, an increase spoke-to-hub arrival time [20], due to the several logistic difficulties, may have delayed the patient’s admission, leading to bad prognosis [15]; as consequence, the mortality among ACS patients markedly increased (9.7% vs. 2.8%) compared to the pre-pandemic era [4].

Despite the reduction in ACS hospitalizations, the severity of clinical presentation was on average higher than in the pre-COVID 19 period. A study, carried out at the Trieste University Hospital [17], highlighted a higher rate of heart failure symptoms at the moment of ACS admission. However, despite the higher number of patients in Killip class ≥ 2 at clinical presentation, the length of hospital stay was not different from the previous years, probably due to hospital’s need to maintain beds availability and to patient’s willingness to be discharged early for the fear of contracting COVID-19. 

The low physical stress and the resting state during quarantine may have reduced the ACS incidence during COVID-19 pandemic; however, the “missed” ACS diagnosis are principally due to a medical care avoidance in COVID-19 era.

## 6. Heart Failure Hospitalizations

Several studies evaluated the rate of heart failure (HF) hospitalizations during the COVID-19 pandemic; a reduction ranging from 49% [21] to 58% [22] has been reported. 

Despite this reduction, a higher prevalence of high-risk features at admission (higher BNP values, lower left ventricular ejection fraction, lower estimated glomerular filtration rate) with a higher number of patients in need of intensive care units (ICUs) have been shown [21].

A similar trend was shown by a multicenter study including eight Italian hospitals [23], which confirmed the reduction in hospitalizations in the period between 21 February and 31 March 2020 compared to intra-year and inter-year control periods, with a higher prevalence of patients with high-risk features (higher NYHA class, lower LVEF). 

An observational, retrospective study in the Tuscany region [22] showed a marked reduction in Emergency Department admissions for cardiac causes after the COVID-19 outbreak with a 58% drop in HF hospitalizations. However, no significant difference in the number of patients in need of ICU admission has been shown (*p*-value = 0.11).

As suggested by the Heart Failure Society of America [24] and European Society of Cardiology (ESC) [25], which strongly suggest the telemedicine system (TMS) use for HF management during COVID-19 pandemic, several hospitals implemented TMS. A recent study including 103 patients showed that 60% were followed up with TMS, and in half of cases its use led to therapy modification; these data underline the role of TMS as a tool to optimize the follow-up of HF patients, especially in the COVID-19 era [26].

## 7. Adherence to Pharmacological Therapy

An observational study, based on the data sourced from the current administrative electronic archives available at Italian Local Health Units (LHUs) participating at Italian Health–DB project [27], reported a high proportion of failed refill of both lipid-lowering drugs (42.4% vs. 36.8%) and biologic therapies (42.5% vs. 33.6%), more likely when the most restricted measures (i.e., lockdown throughout the territory) were applied. 

An observational study reported an 8.5% temporary interruption of the Proprotein Convertase Subtilisin/Kexin type 9 (PCSK-9) therapy for a mean period of 65 ± 1.5 days in patients with dyslipidemias treated with PCSK9 inhibitors (PCSK9i) who missed the cardiologic follow-up visit during the first lockdown. The non-adherent patients showed a marked increase in low-density lipoprotein cholesterol (LDL-C), and 82% of patients moved out of the LDL-C therapeutic range [28]. Probably the prescription methods, the drug availability exclusively by hospital pharmacies, and the need of the pharmacist to communicate the delivery to the Italian Medicines Agency (AIFA) to allow subsequent prescriptions have certainly represented a barrier to PCSK9i access in the health emergency period.

## 8. Analysis of Healthcare System Response to the Pandemic: Lights and Shadows

The impact of the COVID-19 pandemic on Italian Healthcare was characterized by a decrease in both hospitalizations and interventional procedures for cardiovascular diseases, in line with the trend of other European Countries, despite the different capacities and local policies about restriction in hospital admission [29,30,31,32]. 

There are several reasons explaining the reduction in CVD hospitalization in Italy. First of all, the strict rules adopted by the Italian government to contain the spread of the COVID-19 pandemic limited the hospital admission to urgent conditions; the outpatient medical evaluations were stopped, compromising both primary and secondary cardiovascular prevention. Moreover, some patients who required urgent treatment did not refer to hospital or delayed hospital admission, worsening their clinical conditions at presentation, for the fear of in-hospital viral contagion [33].

During the COVID-19 pandemic, the response to non-COVID-19 related emergency was delayed, with longer time in the transfer between spoke-to-hub structures [15,20] and late revascularizations [19]; this may have impacted the patients’ prognosis, since the interval between symptoms’ onset and first medical contact is one of the main predictors of mortality and serious complications in ACS patients [34]. During the first pandemic wave, an increased CVD mortality has been shown among both the general population and ACS patients compared to the pre-pandemic period [4].

The Italian Healthcare System experienced a reallocation of resources and personnel, as a response to this unexpected pandemic, with negative implications in the management of other diseases; these conditions caused indirect and collateral damages on cardiovascular assistance. On the other hand, pre-existing cardiovascular disease or cardiac involvement due to COVID-19 often influenced hospital course and prognosis of COVID-19 patients [35]. So, new protocols for CVD management in COVID-19 patients were needed in the pandemic era. For example, given the high prevalence of atrial fibrillation (AF) during COVID-19 hospitalizations, it was necessary to adjust protocols for AF management (rhythm control drugs, rate control drugs, anticoagulant therapies) on the basis of pharmacological interactions with experimental COVID-19 therapies [36,37].

The increased TMS use helped the healthcare personnel to select cases in need of hospital care, avoiding direct physical contacts in order to reduce the risk of COVID-19 transmission and ensuring continuity in medical assistance pathways [38,39]. Moreover, remote monitoring (RM) aided out-of-office follow-up of CIED patients, evaluation of HF patients, and therapeutic optimization as the adjustment of oral anticoagulant (OAC) dosage based on renal function and/or on patient’s age [40]. TMS and RM are considered in the ESC guidelines as tools to optimize the follow-up of patients affected by HF and those with CIEDs [41]. However, the lack of adequate reimbursement and RM sharing in patients with CIEDs are major obstacles to the implementation of TMS in clinical practice. 

In conclusion, the COVID-19 pandemic offered us the opportunity to understand the limits of the Italian Health service in managing a sudden and unexpected health emergency and the surplus value of telemedicine in the patient’s follow-up, even in the post-COVID 19 period [42].

## 9. Limitations

This narrative review presents some limitations. First of all, it provides a national overview without data subdivision on the basis of the different Italian regions. So, it is not possible to correlate between the reduction in hospitalization rate reduction and the epidemiological pressure across the different Italian regions. Moreover, the overview is focused on the first pandemic wave leaving out a comparison with data relating to successive pandemic waves, which would have provided an analysis of healthcare long-term response to pandemic. Therefore, further analyses will be needed to investigate these aspects.

## 10. Conclusions

The first wave of the COVID-19 pandemic has been responsible for a drastic reduction in the hospitalizations and interventional procedures for cardiovascular diseases, both elective and urgent. The rate of PM implantations for syncope and of CIEDs replacements remained stable compared to the pre-pandemic period. The increased use of TMS and CIEDs remote monitoring helped us to assure continuous care and to reduce the SARS-CoV-2 exposure for staff and physicians. The reallocation of healthcare resources led to delays in the management of time-dependent cardiovascular disease with severe prognostic impact, including a higher CV mortality during the COVID-19 pandemic than previous years.

## Data Availability

Data available in a publicly accessible repository, the data presented in this study are openly available in PubMed.
